# Nasal Septal Agenesis and Attenuated Lower and Upper Lateral Cartilages in a 5-Year-Old Child: A Sporadic Finding

**Published:** 2016-12-28

**Authors:** Khalid Murrad, Faris Aldaghri, Mohamed Amir Mrad

**Affiliations:** ^a^King Saud University, Riyadh, Saudi Arabia; ^b^King Faisal Specialist Hospital and Research Centre, Riyadh, Saudi Arabia

**Keywords:** nasal, septal, agenesis, sporadic, columella

## Abstract

**Introduction:** Cartilaginous nasal septal agenesis is a rare finding. In fact, just one case has been reported to have congenital agenesis of all nasal cartilages in a 6-year-old child by Bakhshaee et al. The literature review shows another case that was reported by Ozek et al in Turkey, where they reported a case of total nasal agenesis that was associated with Tessier no. 30. We could not find a similar case in the literature where only agenesis of the nasal cartilaginous septum was present. **Methods/Case Report:** This is a case report of a 5-year-old child presenting to our clinic with agenesis of his nasal septum and attenuation of the upper and lower lateral cartilages. His parents were seeking a corrective procedure to improve the shape of his nose. He was a male child with a right unilateral cleft lip and palate that were corrected surgically in 2009 (lip repair) and 2010 (palate repair), respectively. **Results:** On postoperative week 3, the patient's mother brought him to the emergency department with a history of falling on his face while playing at home. Examination revealed swelling of the nose but no breathing difficulties. He still had an acceptable augmented nose but with sings of deviation and collapse. **Discussion:** We report this case to find an answer to how such cases can be approached in the future in terms of the surgical intervention required and to study the options of redoing such cases.

Cartilaginous nasal septal agenesis is a rare finding. In fact, just one case has been reported to have congenital agenesis of all nasal cartilages in a 6-year-old child by Bakhshaee et al.^1^ They presented a case of a 6-year-old girl with clinical and radiological findings of agenesis of all nasal cartilages (the quadrangular septum, upper lateral cartilage, and lower lateral cartilage). The literature review shows another case that was reported by Ozek et al^2^ in Turkey, where they reported a case of total nasal agenesis that was associated with Tessier no. 30. We could not find a similar case in the literature where only agenesis of the nasal cartilaginous septum was present.

## METHODS/CASE REPORT

This is a case report of a 5-year-old male child presenting to our clinic with agenesis of his nasal septum and attenuation of the upper and lower lateral cartilages. His parents were seeking a corrective procedure to improve the shape of his nose. He was a male child known with a right unilateral cleft lip and palate that were corrected surgically in 2009 (lip repair) and 2010 (palate repair), respectively. He had no other medical problems, no family history of similar conditions, and had undergone no surgical procedures to his midface other than those mentioned earlier. There was no history of facial trauma, and the patient had no known allergies. No other congenital anomalies were present, and he never suffered from any infections of the face.

No breathing difficulties were reported. The nasal deformity was causing him stress at the school, and it had a great impact on his social behavior.

**Figure 1:**
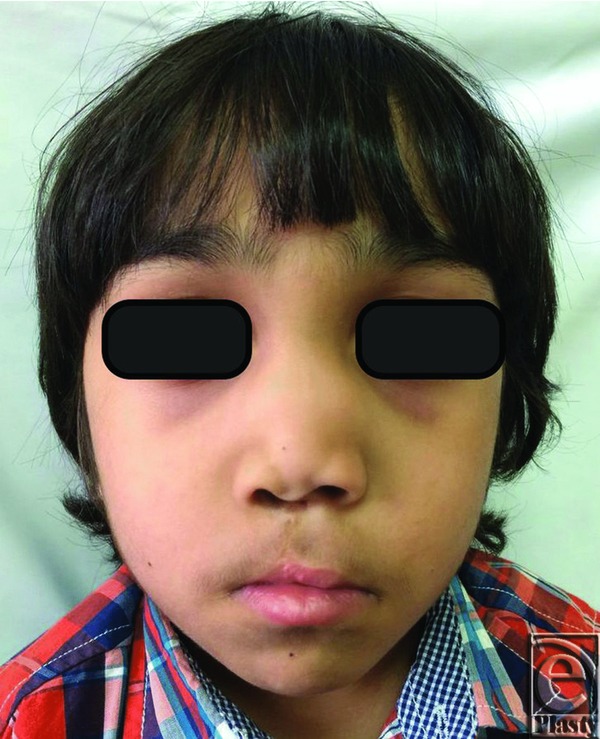
Preoperative frontal view.

**Figure 2:**
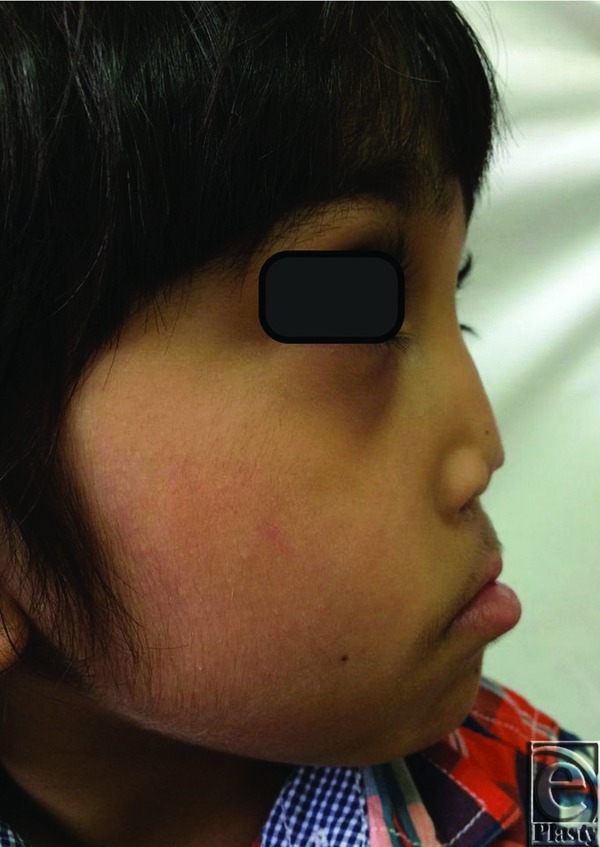
Preoperative lateral view with an apparent collapse of the nose and flattening of the nasal dorsum.

**Figure 3:**
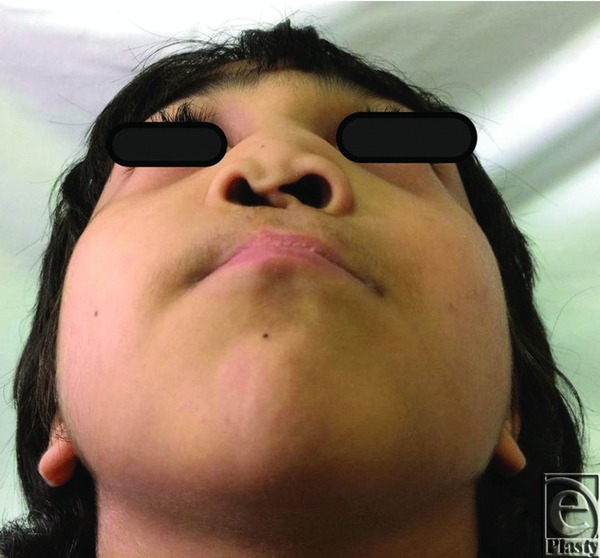
Preoperative base view of the nose with obvious deviation of the columella to the right side and discrepant nostrils.

His physical examination revealed an otherwise healthy child with a paramedian scar on his lip from previous surgery and an obviously collapsed nose in terms of projection with a deviated columella to the right side and discrepancy of the nostrils, with the left nostril being bigger than the right one. His nose was rotated downward, and he also had a short, thin, and curved columella. His nasolabial angle was 80° and his nasofrontal angle was 160° (Figs 1-3). His intranasal examination showed no septal support, and his right internal valve was collapsed. His speech showed hypernasality. The remainder of his physical examination was negative for abnormal midface deformities or malocclusion.

A preoperative computed tomographic (CT) scan of the facial bones was obtained to assess his nasal bone structure and position. CT scans showed flattening of the nasal bones and the presence of the bony septum. Nasal cartilages were not visible.

## OPERATIVE PROCEDURE

After obtaining surgical consent from the family, the patient was taken to the operating room and the senior author (M.A.M.) decided to correct the deformity with a rib bone and cartilage graft. During the dissection, we were unable to identify any cartilaginous septum and his lower and upper lateral cartilages were attenuated. In addition, the medical crura of the lower lateral cartilages were entirely missing.

**Figure 4:**
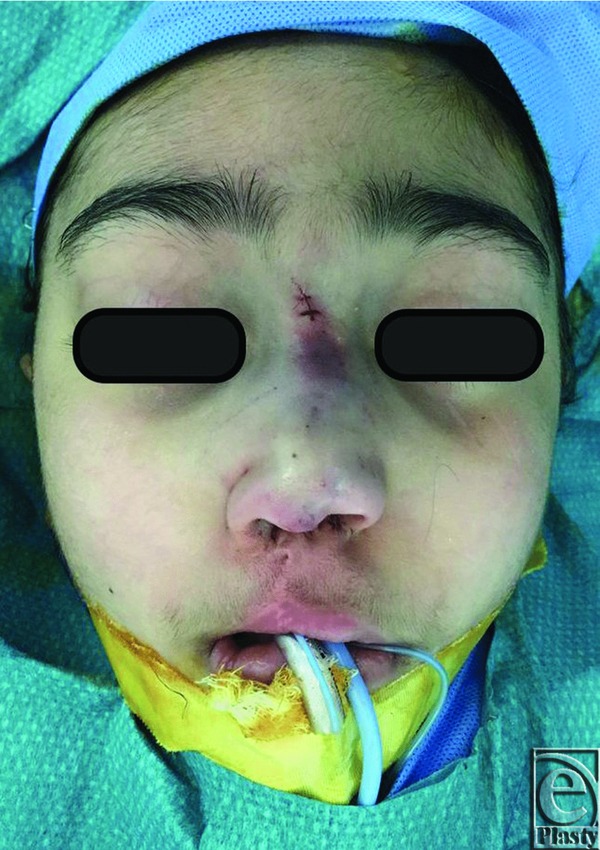
Intraoperative frontal view of the patient after placement of the rib cartilage graft.

**Figure 5:**
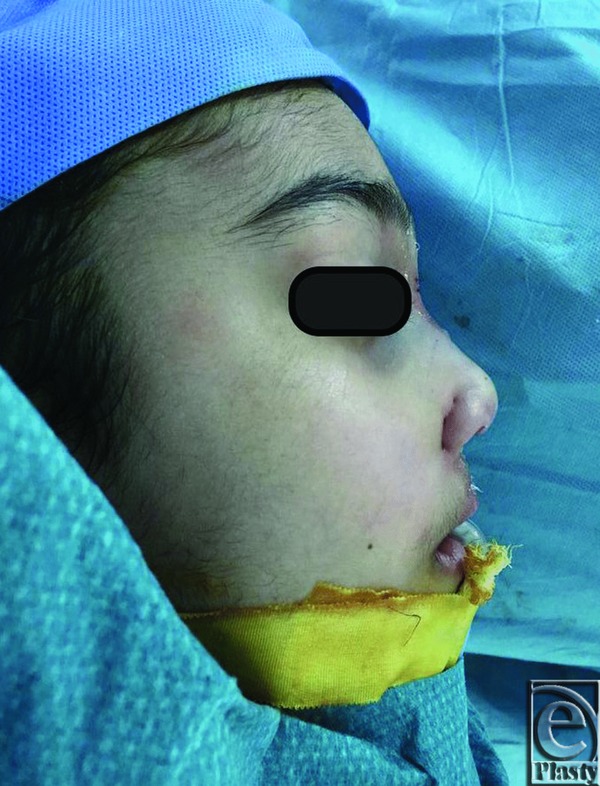
Intraoperative lateral profile after placement of the rib cartilage graft, with a remarkable positive gain in the nasal dorsum definition.

**Figure 6:**
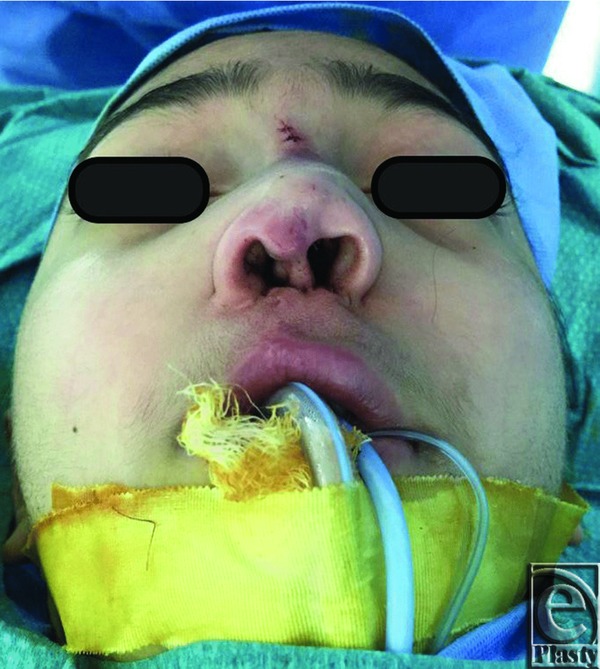
Intraoperative base view after correction with a noticeable change in the nasal collapse and a resultant columellar straightening.

A 6-cm rib bone and cartilage graft (1-cm bone and 5-cm cartilage) was harvested from the right fifth rib. Through a left intercartilaginous incision, the osseous end of the graft was fixed to the nasal bones using 2 percutaneous screws. With this maneuver, we achieved dorsal nasal augmentation while also correcting the collapsed nose (Figs 4-6). Because of the thin layers of mucosa in the septal area, we decided not to insert a columellar strut. The incision was closed using 4-o Vycryl rapid. The nose was then covered with Steri-strips, and a nasal splint was applied. The postoperative hospital stay was uneventful. The patient was seen in the clinic 1 week postoperatively for a routine follow-up, and both the patient and his family were satisfied with the nasal augmentation and nasal collapse correction.

## RESULTS

**Figure 7:**
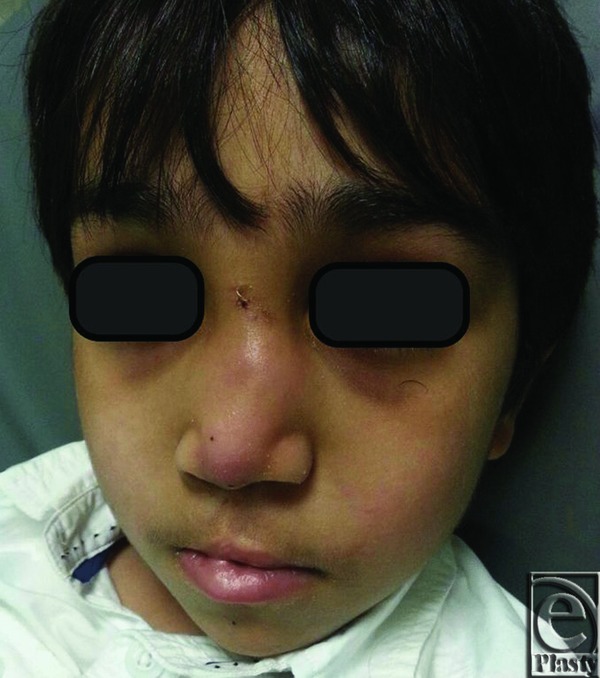
Upon presentation to the emergency department with a noticeable swelling of the nose and minimal deviation of the dorsum of the nose.

**Figure 8:**
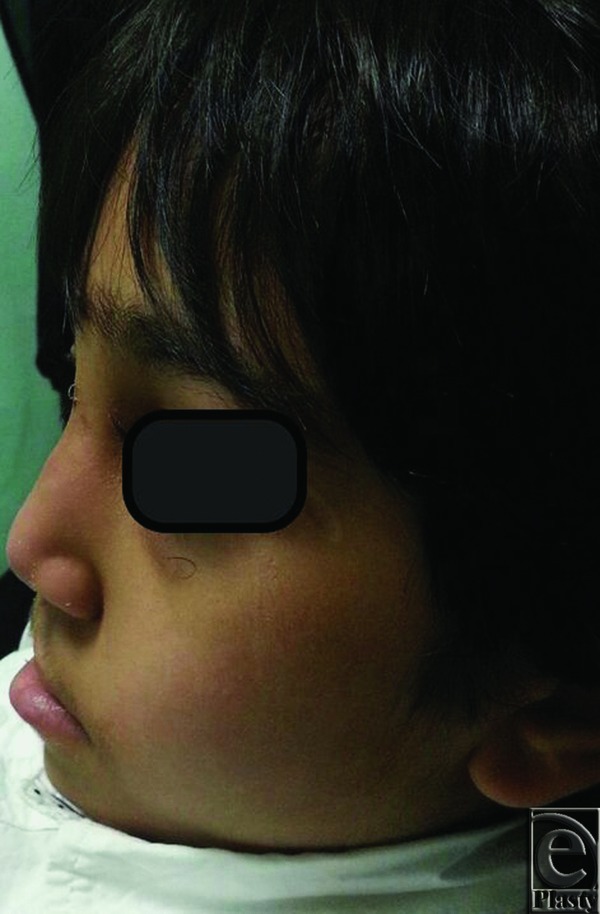
A lateral profile of the patient showing a minor collapse of the nasal dorsum.

**Figure 9:**
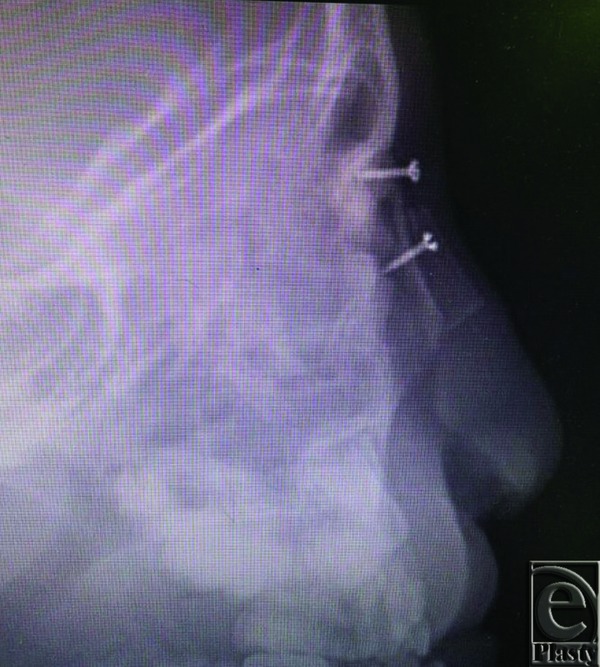
X-ray film showing the position of the screws used for fixation of the graft.

**Figure 10:**
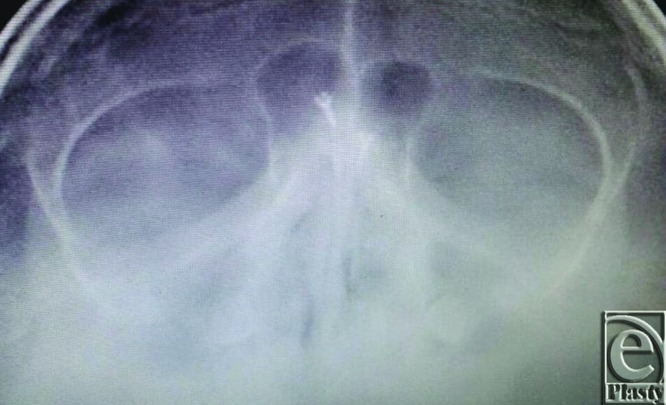
Another view showing the screws in place with no signs of dislodgment.

On postoperative week 3, the patient's mother brought him to the emergency department with a history of falling on his face while playing at home (Figs 7 and 8). The examination revealed swelling of the nose but no breathing difficulties. Clinical and radiological examinations showed that the rib graft had broken at its weakest point, the osseocartilaginous junction (Fig 9 and 10). He still had an acceptable augmented nose but with sings of deviation and collapse. The patient was given an appointment after 4 weeks to decide on the next corrective surgery.

## DISCUSSION

Our case patient had a history of cleft lip and palate with clinical identification of the absence of the nasal septal cartilage but the presence of the lower lateral cartilage, though, deformed and attenuated. No genetic workup was done for the case patient, although it would be interesting to see if this finding has any genetic association. We appreciate the presence of midfacial retrusion, although this can be explained by the effect of the palatoplasty that was performed earlier.^3^ Holoprosencephaly (HPE) was among our differential diagnosis. As it is known that HPE has various presentations depending on the severity, the mild form presents with no brain malformation but other craniofacial malformations, such as cleft palate and nasal deformities including nasal septal agenesis, are present.^4^ Our case did not fit the usual presentation of HPE, not even in its mild form.

We report this case to find an answer to how such cases can be approached in the future in terms of the surgical intervention required and to study the options of redoing such cases. In addition, we could not relay this finding to any embryological abnormality that would result only in the deficiency of the cartilaginous nasal septum. Robinson and Hilger^5^ described a case of Binder syndrome that is characterized by the regression of the midface structures and anomalous (hypoplastic) cartilages including the nasal cartilages.
